# Dynamic model of whole cortex reveals disassortative hub structure in the intracortical connectome

**DOI:** 10.1186/1471-2202-16-S1-P57

**Published:** 2015-12-18

**Authors:** Matthieu Gilson, Ruben Moreno-Bote, Adrian Ponce-Alvarez, Petra Ritter, Gustavo Deco

**Affiliations:** 1 Center for Brain Cognition, Universitat Pompeu Fabra, 08018, Barcelona, Spain; 2 Parc Sanitari Sant Joan de Déu and Universitat de Barcelona, Esplugues de Llobregat, 08950, Barcelona, Spain; 3 CIBERSAM, Esplugues de Llobregat, 08950 Barcelona, Spain; 4 Bernstein Center for Computational Neuroscience, Berlin, 10117, Germany

## 

The brain exhibits complex spatio-temporal patterns of activity. In particular, its baseline neural activity for idle subjects or animals has a specific structure, also called functional connectivity (FC): cortical areas experience correlated fluctuations at rest as revealed by imaging techniques (e.g., fMRI, EEG and MEG). Moreover, these correlation patterns become more expressed or suppressed depending on the engaged task. The putative function for this stereotypical background activity has been actively questioned by the neuroscience community. A Bayesian hypothesis is that it reflects the variety of possible coactivations of cortical areas for usual tasks, forming a prior representation of the environment and interactions wherewith.

The present study relies on our recently developed model [1] in which noise diffusion is constrained by intracortical white-matter projections to reproduce FC observed in experimental data (fMRI BOLD signal). In this model, noise has a functional role and represents the variability of neural activity during tasks. This model allows us to explore the role of various parameters in shaping FC, such as the local nonlinear dynamics of cortical areas. More importantly, we can infer the efficacies of intracortical projections, also called effective connectivity (EC), from experimental data. EC plays a crucial role in shaping FC and differs from the structural connectivity (SC) that is typically obtained using diffusion tensor imaging (DTI), which estimates the density of white-matter fibers. It is important to notice that the efficacies of these fibers may differ from their density, due to the concentration of synapses formed at their end, the type of neurotransmitters associated and the excitability of target neural populations. In the end, our model combines anatomical SC and activity FC to evaluate what drives the neural dynamics, embodied in EC.

Practically, directed EC can be estimated using two matrices of empirical FC, namely FC0 for correlations with zero time shift and FCτ for correlations with a given time shift τ>0. We apply our method to infer the intracortical EC from experimental fMRI data that consists of ~20 minutes of recording for 25 idle subjects. The model reproduces both empirical FC0 and FCτ matrices with a Pearson correlation coefficient of 0.65. The recovered EC connectivity is significantly asymmetric, with a value of 0.3 for an index that scales from 0 for symmetric matrices to 1 for asymmetric matrices. Moreover, EC exhibits hubs that have either strong incoming or outgoing weights, but not both. This disassortative property is expected to exert a strong influence in shaping the whole network dynamics.
